# Bibliographical Notices

**Published:** 1839-08-31

**Authors:** 


### BIBLIOGRAPHICAL NOTICES.

*An Experimental Inquiry into the Physiology of Cutaneous Absorption, and its application to Therapeutics.* By WILLIAM HERRIES MADDEN, M. D., Licentiate of the Royal College of Surgeons, etc. Edinburgh: 1838. 8vo. pp. 152.

THE doctrine of cutaneous absorption includes the therapeutic effects of medicinal baths, a means of treatment of great value in the management of both acute and chronic disease. It is difficult to say why this therapeutic agent (with the exception of sea-bathing) should be so needlessly neglected both in this country and in Great Britain; it is free from many inconveniences that attend the administration of internal remedies in a number of disorders, and sometimes has a physiological connexion with the disease which enables us to act much more directly upon it, than we can do by any internal medicine. In our own experience we have long estimated the importance of medicated baths at a very high standard, and we have rarely been disappointed in the good results we anticipated from their employment.

The diseases of children are very advantageously treated by these baths. As a tonic, the warm or cold salt bath is most useful; as an alterative, none answers so well in the large majority of cases as the sulphur bath, which is formed artificially by dissolving the sulphuret of potassa in water. The good effects of the sulphur bath as an alterative in the diseases of children, became very manifest at the hospital at Paris, which is specially appropriated to them. The usual mode of treating children affected with itch, is to place them in a course of sulphur baths, which are given five times a week; two always are omitted on account of the domestic arrangements of the hospital. The children subjected to this course almost universally become fat, and enjoy much more robust health than at the time of their admission. From this circumstance the remedy was introduced into the treatment of several diseases bearing no direct connexion with the skin. Amongst them were chorea and chronic diarrhœa, which is so frequent a sequela of the exanthematous diseases of children. In both these diseases the success of the sulphur bath, as a general modifier, was most remarkable, and became part of the established practice of the institution. The use of iodine, administered in the same way, is long known, and duly appreciated by practitioners; and the number of medicinal agents used in this way is undoubtedly susceptible of very great extension.

The cutaneous absorption of medicines is by no means confined to them, as administered in the form of baths. Any mode of application which either softens the cuticle by the aid of a liquid, or long-continued friction, or which consists in the direct application of the medicine to the skin, after previously removing the cuticle, will produce absorption. That is, provided the remedy be soluble, and not productive of much immediate irritation of the skin.

The fitness of medicinal substances for cutaneous absorption, is, however, somewhat variable, and may depend upon some peculiar property unconnected with solubility. Thus, it will be found that opium and belladonna are much more active in a proportionate dose than some other narcotics; and that aloes and gamboge are most active as purgatives. The difference may, however, depend, in part, upon the ready solubility of the remedy in the fluids of the body, the smallness of the dose at which the remedy proves active. If the necessary dose be large, the extent of surface acted upon is often disproportionately small. Narcotics, as a general rule, act more powerfully than any other class of substances; we may, in this case, admit, that they exercise a local action upon the nerves which propagates itself in a reflex manner to the great nervous centres, and that their power does not altogether depend upon the absorption.

Dr. Madden has given a condensed account of the observations hitherto made upon this subject, and has added a few new experiments of his own, which illustrate the action of baths in favouring the absorption of medicinal substances by the skin. A part of his work contains the description of other methods of introducing medicinal substances by the skin, such as that of frictions, as proposed by Chrestien of Montpellier, and the endermic method. We were somewhat disappointed in not meeting with a larger number of original facts, particularly connected with the treatment of disease by internal medication; but, though there is little positively new in the work, what is already known is classed in a clear and methodical manner.

---

The Lectures of Sir Astley Cooper, Bart., F. R. S., *Surgeon to the King, &c., on the Principles and Practice of Surgery, with additional Notes and Cases.* By Frederick Tyrrell, Esq., Surgeon to St. Thomas's Hospital, and to the late London Ophthalmic Infirmary. Fifth American, from the last London Edition. Complete in one Volume. Philadelphia: 1839. pp. 580, 8vo. Haswell, Barrington & Haswell.

We are glad to see that the importance attached to the lectures of Sir Astley Cooper, calls for a fifth edition. This work is illustrated by two plates, showing the position of the bone in dislocations of the hip-joint, and the mode of effecting its reduction. The diseases of the breast are shown in two plates containing drawings of several diseased specimens, some of which are coloured. From the great expense of the large works on dislocations, hernia, &c., they are necessarily inaccessible to the large majority of practitioners, so that the republication of these lectures, embodying the more important points on these subjects, is particularly valuable.

From the extensive hospital and private practice enjoyed by the lecturer during a period of near fifty years, his opinions on all subjects relating to surgery are of peculiar importance; and his lectures may be regarded equally necessary to practitioners who devote themselves more immediately to the practice of surgery, and to those who, not making that branch the object of peculiar attention, may stand in need of a work of reference.

Dr. Alexander Lee, of London, is now editing a complete set of Sir Astley Cooper's works, in octavo, with all the plates in reduced size. Two volumes of the work have already appeared; the remaining volume will be soon forthcoming.

